# The Link between Periodontal Disease and Asthma: How Do These Two Diseases Affect Each Other?

**DOI:** 10.3390/jcm12216747

**Published:** 2023-10-25

**Authors:** Hiroyuki Tamiya, Masanobu Abe, Takahide Nagase, Akihisa Mitani

**Affiliations:** 1 Division for Health Service Promotion, The University of Tokyo, 7-3-1 Hongo, Bunkyo-ku, Tokyo 113-0033, Japan; 2 The Department of Respiratory Medicine, The University of Tokyo Hospital, 7-3-1 Hongo, Bunkyo-ku, Tokyo 113-8655, Japan; 3 Department of Sensory and Motor System Medicine, Graduate School of Medicine, The University of Tokyo, Tokyo 113-0033, Japan

**Keywords:** asthma, gingivitis, inhaled medication, oral health, periodontitis

## Abstract

A growing body of evidence suggests that the effects of poor oral hygiene extend beyond the oral cavity and are associated with a variety of systemic diseases, including asthma. Asthma, which results in symptoms of cough, wheezing, and dyspnoea, and is characterized by airflow limitation with variability and (partial or complete) reversibility, is amongst the most prevalent respiratory diseases with approximately 262 million patients worldwide, and its prevalence and disease burden is on the increase. While asthma can occur at a young age, it can also develop later in life and affects a variety of age groups. Both of these diseases have a chronic course, and various researchers have suggested a link between the two. In this article, we aim to provide a literature review focusing on the association between the two diseases. The results demonstrate that medications (primarily, inhaler medicine), hypoxia induced by asthma, and the breathing behaviour of patients potentially trigger periodontal disease. In contrast, oral periodontopathogenic microorganisms and the inflammatory mediators produced by them may be involved in the onset and/or exacerbation of asthma. Common contributing factors, such as smoking, gastro-oesophageal reflux, and type-2 inflammation, should also be considered when evaluating the relationship between the two diseases.

## 1. Introduction

Poor oral hygiene is common in many countries worldwide and can trigger not only oral diseases, but also systemic diseases, leading to a significant health burden [1]. Among these, periodontal disease is frequent, and numerous studies have been conducted on their association with systemic diseases. Periodontal disease consists of gingivitis and its advanced form, periodontitis. It is a chronic inflammatory disease caused by the bacterial infection of the periodontium and leads to the dissolution of the surrounding structure, including the gums around the teeth and the bone that supports the teeth. If the border between the teeth and gums (gingival sulcus) is not cleaned properly, a large number of bacteria can grow there, causing inflammation of the gingival margins [2]. Periodontitis affects about 50% of the population worldwide [3], and severe periodontitis is found in 9.8% of the global adult population [4]. Severe periodontitis ultimately causes masticatory dysfunction and nutritional compromise, aesthetic impairment, altered-speech, low self-esteem, and a poorer overall quality of life (QOL) [5].

Asthma is characterized by a chronic inflammation of the airways that causes respiratory tract hyperresponsiveness to various stimuli, resulting in the narrowing of the airways [6]. According to the World Health Organization, asthma affected an estimated 262 million people in 2019 and caused 455,000 deaths [7]. The frequency of comorbidities is increased in older adult patients with asthma [8]. These comorbidities can worsen respiratory symptoms, decrease QOL, and worsen asthma control [6]. In Japan, approximately 90% of deaths due to asthma occur in adults aged 65 years or older [9].

In recent years, considerable attention has been paid to the interaction between oral and systemic diseases, including asthma. Questionnaire surveys indicate an association between oral diseases, such as periodontal disease and malocclusion, and asthma [10,11]. It is important in healthcare to understand the relationship between oral diseases and systemic diseases and whether interventions for oral disease can improve asthma prognosis and extend healthy life expectancy rates, especially in an ageing society [12,13]. Nevertheless, conflicting reports of these associations exist, and the underlying biological mechanisms are not yet fully understood. This review aims to provide suggestions on the possible interactions between periodontal diseases and asthma, the impact of therapeutic agents, and the directions for future research.

## 2. Asthma and Periodontal Disease

### 2.1. Periodontal Disease and Its Impact on Asthma

Some cross-sectional studies suggest an association between asthma and periodontal disease. Gingival bleeding was correlated with asthma in late adolescents by questionnaire surveys in Japan [11]. A self-reported allergic disease diagnosis, including asthma, showed a positive correlation with poor oral health, including periodontal disease in adolescents, in the Korean national database survey [14]. In this study, the adjusted odds ratio (OR) for asthma was 1.48 in the poor oral health group compared to the group with good oral health. Another Korean, nationwide, population-based survey revealed that participants with a current asthma condition were five-times more likely to have periodontal disease (adjusted OR, 5.36) than those without it [15]. Wee et al. also reported a higher prevalence of asthma in the poor oral hygiene group than in the good/normal groups. Subjective poor oral hygiene was significantly associated with asthma, with an adjusted OR of 1.19 [16]. Brasil-Oliveira et al. assessed the oral health-related QOL (OHRQoL) among individuals with severe asthma. Periodontitis was more common in patients with severe asthma than those with mild-to-moderate asthma and those without, and negatively impacted on their OHRQoL [17].

Case-control studies have also described a significant association between periodontal disease and asthma. McDerra et al. conducted a comparison of gingivitis and plaque scores between children with asthma aged 4–16 years on inhaler therapy and children without asthma matched for age, sex, race, and socioeconomic status. They reported that asthmatic children had more severe gingivitis and plaque deposits [18]. Gomes-Filho et al. reported that adult patients with periodontitis were 4.82-times more likely to have severe asthma than those without [19]. Soledade-Marques et al. compared the prevalence of periodontitis between severe asthma and control groups and found that individuals with periodontitis had about a threefold increased risk of severe asthma than those without periodontitis [20]. Lopes et al. also showed a positive association between periodontitis and severe asthma with an OR of 4.00, and presented a higher level of *Prevotella intermedia*, a periodontitis-related oral pathogen, in the severe asthma group compared to the control group [21]. Patients with asthma in Jordan were three-times more likely to have periodontitis than the controls, and the extent and severity of periodontal disease were significantly higher in patients with asthma. The risk of periodontitis and clinical attachment loss (CAL) ≥ 3 mm were higher in patients on oral corticosteroid treatment compared to inhaled corticosteroids (ICSs) [22]. In a study in India, periodontal indices, including the plaque index (PI), calculus index, gingival index (GI), and papillary bleeding index measurements, were higher in the asthma group than the control group. Moreover, all of these parameters were higher in patients with moderate-to-severe asthma than in those with mild asthma [23]. Similar results were obtained by another group [24,25].

In the population-based retrospective cohort study in Taiwan, patients with asthma were at a 1.18-fold elevated risk of developing periodontal diseases. The risk is much higher for those with emergency medical needs, hospitalisation, and those undergoing ICS treatment [26]. An association between asthma and periodontal disease was also suggested in a population-based prospective cohort study conducted in seven northern European centres. The authors demonstrated that the presence of asthma and asthma severity assessed by the number of symptoms were associated with frequent gingival bleeding [27].

Table 1 summarizes the supportive observational studies on the association between periodontal disease and asthma. Since the adjusted confounders vary among studies and prospective investigations are limited, further research is needed to determine the causal relationship between the two.

The association was also demonstrated in several meta-analyses. Moraschini and colleague performed a meta-analysis of 21 studies examining the relationship between asthma and periodontal disease and found that patients with asthma showed poorer oral health (as assessed by gingival bleeding, PI, and GI parameters) compared with healthy individuals [28]. The meta-analysis conducted by Ferreira et al. showed higher mean values for the calculus index, papillary bleeding index, and CAL in the asthma group, although the differences for GI and PI were not statistically significant [29]. Gomes-Filho et al. showed an association between periodontitis and asthma in a meta-analysis of three studies, with an adjusted OR of 3.54 [30]. Wu and colleagues demonstrated a significant correlation between pulmonary and periodontal diseases, with a pooled adjusted OR of 3.03 for asthma. The study showed that CAL, PI, and GI were lower in patients with asthma, indicating poorer periodontal health [31].

### 2.2. Effectiveness of Periodontal Interventions

It is important to assess whether oral care interventions improve asthma symptoms and other outcomes to determine if there is a link between the two.

Whether an intervention for oral disease can improve asthma-related outcomes is still under debate. Shen et al. demonstrated that adult patients with asthma undergoing periodontal treatment had a lower incidence of hospitalisation due to respiratory events than those without periodontal disease by the propensity score-matching method using the nationwide database [32]. They also showed a lower all-cause mortality rate in the periodontal treatment group. These results, along with the importance of intervening in periodontal disease, suggest that there may be unrecognized periodontal disease in patients with asthma. Enomoto et al. investigated the effect of withholding dental care visits due to behavioural restrictions caused by coronavirus disease 2019 on the control of systemic diseases. The results of their study revealed that patients who discontinued dental treatment experienced significantly more asthma exacerbations than those who continued (27.8% vs. 11.5%) [33].

Pambudi et al. investigated the effect of periodontal treatment for respiratory quality in asthmatic children by a randomized, parallel group trial. They showed a reduced rate of Gram-negative bacilli colonization in the dental plaque after periodontal treatment, and its association with improved asthma QOL, airway reversibility, and decreased number of blood eosinophil [34]. Nelwan et al. investigated the impact of scaling and root planning on the level of serum immunoglobulin (Ig) E and IgG4 in children with gingivitis and a house-dust-mite allergy. Most children had concurrent asthma. The results of this study suggest that 6-month comprehensive dental scaling combined with root planning may help to reduce IgE levels in this population [35].

There are a limited number of studies examining the effect of oral care on asthma outcomes in prospective trials, and the number of participants is low (Table 2). Further studies are warranted to draw adequate conclusions.

### 2.3. Discrepancies in the Data

Although many studies suggest a link between asthma and poor oral hygiene, some controversy remains. The data from the Third National Health and Nutrition Examination Survey (NHANES) III (1988–1994) in the United States, including 1596 adolescents who had an asthma diagnosis, were analysed for various periodontal measures, including bleeding on probing, subgingival calculus, supragingival calculus, a probing depth greater than or equal to 3 mm, and a loss of periodontal attachment greater than or equal to 2 mm, none of which were associated with asthma severity or with the use of anti-asthmatic drugs [36]. Using the NHANES database, Shah et al. examined the association between asthma and periodontitis in the adult population. They showed that patients with asthma were significantly less likely to have severe periodontitis [37]. Chatzopoulos and colleagues found that asthma was significantly associated with the decreased risk of radiographically confirmed bone loss, which reflected the severity of periodontal disease, with an OR of 0.695 using the dental record database in the United States, although the data on individuals’ medications were lacking [38]. Lemmetyinen et al. conducted a population-based matched cohort study and did not find significant associations between dental diseases (including tooth decay, chronic apical periodontitis, sialadenitis, and diseases of the periodontal tissue) and asthma in the adult population using the national hospital discharge registry in Finland [39]. Hozawa and colleagues found that periodontal disease was associated with a decreased risk of asthma exacerbations using the Japanese claims database [40]. They speculated that patients with asthma who received sufficient doses of ICS may have achieved better disease control and had fewer exacerbations, while their oral hygiene may have been compromised by the high amount of ICS.

Ho et al. point out that allergic rhinitis (AR), which is often comorbid with asthma, may be a confounding factor. They investigated the association between the five major oral diseases (dental caries, periodontitis, pulpitis, gingivitis, and stomatitis/aphthae) and the presence of AR and asthma using a national database in Taiwan. The incidence of all five oral diseases was higher in the AR group than in the non-AR group after an adjustment for confounders and asthma. Similar findings were observed in the asthma group when not adjusted for AR. However, when adjusted for AR, they found no association between asthma and oral disease [41].

A recent meta-analysis found no significant association between periodontal disease and asthma [42].

Under the ‘hygiene hypothesis,’ some researchers have suggested that periodontal disease may have a suppressive impact on the susceptibility to allergic disease [43]. Within this framework, it is presumed that the microbial colonization in the body would trigger a systemic reaction to prevent the development of allergic disease. Cross-sectional studies revealed that the elevated serum concentrations of IgG antibodies against periodontal pathogens *Porphyromonas gingivalis* and *Actinobacillus actinomycetemcomitans* were significantly associated with a lower prevalence of asthma and/or wheezing [44,45]. In a study of obese or overweight patients with asthma, the OR for having an asthma diagnosis in participants with severe periodontitis was 0.44 for those with none/mild periodontitis. For participants with severe periodontitis, the OR for receiving asthma medications was 0.20 for those with no/mild periodontitis [46]. Friedrich and colleagues investigated the relation between periodontitis and respiratory allergies in a prospective cohort study, and found an inverse association between periodontitis and hay fever and house-dust-mite allergies. They also uncovered a weak inverse relation between periodontitis and asthma [47]. Similarly, they also reported a strong inverse association between periodontal disease and respiratory allergies (hay fever, house-dust-mite allergy, and asthma) in patients with type-1 diabetes [48]. A study in Australia found that the prevalence of asthma was lower in patients with periodontal disease than in the general population (1.5% vs. 5.6%), although the frequency of allergies (29.2% vs. 22.9%) and pulmonary disorders (8.5% vs. 4.3%) was higher in the periodontal group [49]. When periodontitis was induced in asthmatic mice, inflammatory cells, such as eosinophils, lymphocytes, and macrophages, in bronchoalveolar lavage fluid decreased. In addition, levels of interleukin (IL)-4 and tumour necrosis factor-α were reduced, as was the production of airway mucus [50]. Meanwhile, a recent Mendelian randomization analysis found no causal effect of periodontal disease on the development of asthma, although asthma might be protective against periodontal disease [51]. The relationship between asthma and periodontal disease is complex and influenced by many factors, including used inhaled medications, comorbidities, and socioeconomic status. The association needs to be confirmed by the research that adequately controls for these factors (Table 3).

## 3. How Are Periodontal Diseases and Asthma Related? Biological Evidence

Both the condition of asthma itself and the medications used to treat it can contribute to the development or progression of oral diseases. There also may be common predispositions that make a host more susceptible to both diseases. We discuss below some possible mechanisms that may be involved in the relationship between oral diseases and asthma.

### 3.1. Common Predisposing Factor

In the field of cardiovascular diseases, certain common genetic predisposing factors are known to be a risk factor for both periodontal and cardiovascular diseases. Some examples include gene variants of the angiotensin-converting enzyme and the angiotensin II type-I receptor in hypertension and periodontal disease [52]. Regarding asthma and oral diseases, Loos et al. analysed single nucleotide polymorphisms in the nucleotide binding oligomerization domain protein 1 (NOD1) gene in patients with aggressive periodontitis, genetic polymorphisms known to be associated with inflammatory bowel disease and asthma, but failed to demonstrate an association between them [53]. Although such shared genetic factors are not yet fully elucidated in asthma and oral diseases, certain diseases are known to be triggers for both. For example, gastroesophageal reflux may cause both periodontal disease and asthma. Previous studies have shown that patients with gastro-oesophageal reflux disease (GORD) have an increased risk of gingival inflammation and poor oral hygiene due to gastric acid reflux [54,55]. Liu et al. found an association between the symptoms of severe periodontitis and the risk of GORD (OR, 1.40) using a Chinese nationwide cross-sectional survey [56]. A Korean cross-sectional study revealed that GORD was independently associated with an elevated risk of chronic periodontitis (OR, 2.883) [57]. The risk of developing periodontitis was 1.38-fold higher in patients with GORD than in those without in a study using the Taiwan National Health Insurance Research Database [58]. A Mendelian randomization study also demonstrated the causal effect of GORD on periodontitis (OR, 1.229) [59]. Periodontal pathogens, including *Veillonella*, *Prevotella*, *Porphyromonas*, and *Fusobacterium*, were commonly found in the oesophagus of patients with GORD, suggesting the biological relationship between these two diseases [60].

Simultaneously, GORD has also been suggested to be associated with asthma [61,62]. Meanwhile, some asthma medications, including beta-2 agonists and theophylline, cause the relaxation of the lower oesophageal sphincter [63]; therefore, the effects of the medication need to be interpreted with caution.

The relationship between periodontal disease and type-2 inflammation has also been documented in several reports. The levels of granulocyte macrophage colony-stimulating factor and IL-5, both Th2 cytokines, produced by peripheral blood mononuclear cells from adult patients with periodontitis were comparable to those from patients with allergic asthma, suggesting that the Th2 pathway may be activated in chronic inflammation in both diseases [64]. The authors speculated that these cytokine expressions may contribute to increased susceptibility of the host to periodontal diseases. Type-2 inflammation has also been implicated in the development of a subset of asthma, and hosts predisposed to this type of inflammation may be more likely to develop both asthma and periodontitis. The involvement of innate lymphoid cells (ILCs) in periodontal disease is controversial. While one group stated that ILC2s were the most common of the ILCs in periodontal tissues [65], others reported that ILC1s and ILC3s accounted for the majority of the ILCs in periodontitis or gingivitis tissues [66,67]. Although ILC2 is considered to be mainly involved in the pathogenesis of asthma, some reports suggest the involvement of ILC1 in neutrophilic asthma and asthma–chronic obstructive pulmonary disease (COPD) overlap [68]. Further research on the link between ILCs and periodontal disease is required.

The role of airborne allergens has also been discussed. Son et al. has examined the effect of German cockroach extract, a known exacerbator of asthma, on human gingival epithelial cells. They found that German cockroach extract activated protease-activated receptor-2 and induced inflammation in human gingival epithelial cells via the production of pro-inflammatory cytokines, including IL-1b, IL-6, and IL-8, and the nucleotide-binding oligomerization domain-like receptor family pyrin domain containing 3 [69]. This indicates that gingivitis and asthma exacerbation may be triggered by a common inflammatory process.

IL-9 has been reported to be associated with the development of allergic diseases, including asthma [70], and a recent Mendelian randomization analysis also suggested an association between circulating IL-9 and periodontal disease [71]. However, the evidence is still limited, and more research is needed on this key point.

Cigarette smoking is a well-known factor involved in both asthma and periodontal disease. Cigarette smoking not only leads to the development of asthma [72,73,74], but also to worse clinical outcomes, including a deterioration of symptoms, increased exacerbation frequency, poor QOL, and progression of airflow obstruction [75]. Smoking cessation improves symptom control, airway inflammation, and respiratory function in patients with asthma [76]. Cigarette smoking is also an established risk factor for periodontal disease, resulting in its development as well as a decreased effectiveness of periodontal treatment [77]. ALHarthi et al. analysed the impact of smoking cessation on periodontal disease using the NHANES database. They observed that, in ex-smokers, the OR for periodontitis decreased significantly by 3.9% for each year after quitting smoking after an adjustment for confounders, and concluded that the likelihood of periodontitis decreased with the length of quitting smoking among ex-smokers [78]. Smoking also enriches *Fusobacterium* species in the subgingival plaque in patients with asthma, and this microorganism is associated with the development of periodontal disease [79].

Vitamin D insufficiency may be involved in both asthma and periodontal disease. According to the prospective cohort study using the UK Biobank, individuals with optimal serum vitamin D levels had an 11.1% reduced risk of developing asthma, compared to individuals with a vitamin D deficiency (hazard ratio [HR] = 0.889; 95% CI = 0.820–0.964; *p* = 0.005) [80]. In patients with asthma who had vitamin D insufficiencies, the supplementation of vitamin D was also shown to reduce the risk of asthma exacerbations [81,82]. In a double-blind, randomized, placebo-controlled trial, vitamin D supplementation attenuated eosinophilic airway inflammation in patients with nonatopic asthma [83]. Vitamin D deficiency has also been suggested to be associated with periodontal disease. The meta-analyses revealed lower levels of serum vitamin D or 25-hydroxyvitamin D in patients with periodontal diseases compared to healthy individuals [84,85]. In several randomized controlled trials, vitamin D supplementation was shown to improve the periodontal parameters, including CAL, GI, PI, and PD [86]. These findings suggest that vitamin D insufficiency may negatively affect both periodontal disease and asthma. However, the association between vitamin D levels and periodontal disease is controversial and warrants further research [87,88,89].

It has been suggested that obesity may also be involved in both of the diseases. Several systematic reviews have shown a positive association between being overweight or obese and periodontal disease [90,91,92,93]. Similarly, a link between obesity and the development of asthma has also been suggested [94]. Obesity has also been identified as a risk factor for severe asthma in a subset of patients, particularly those with non-type-2 asthma [95,96]. Adipose tissue-induced low-grade systemic inflammation plays a role in respiratory tract inflammation and the exacerbation of asthma [97]. When investigating the relationship between asthma and periodontal disease, it is important to consider the impact of obesity.

### 3.2. How Do Asthma Medications Affect Oral Health?

#### 3.2.1. Roles of Saliva and Oral Microbiome

The connection between asthma medications and oral diseases should also be discussed. Inhaler medicines play a critical role in the treatment of obstructive airway diseases, including asthma. Of the inhaled dose, only approximately 20% actually reaches the lungs, while the majority remains in the oropharynx [98]. Therefore, drug residues in the oral cavity can introduce a variety of effects, i.e., on the properties of saliva and oral microbiome. Some oral medications can also affect periodontal hygiene (Table 4).

Salivary flow enables mechanical cleaning against food debris or microorganisms, and intraoral clearance is linked to the secretion amount [99]. Due to its properties, saliva is responsible for keeping the pH of the oral cavity at a neutral level [100]. These effects may be compromised by inhaled medications due to the hyposalivation characteristics of these drugs [101,102]. Saliva provides nutrients to the oral microflora, transports antimicrobial factors, and maintains microbial homeostasis [103]. More than 700 bacterial species have been recognized in the oral microflora, with *Firmicutes* being the most frequent bacteria phylum found in human saliva [104,105]. Asthma and its treatment are known to disturb the balance of the oral microbiota and increase pathogenic bacteria. It was found that the family *Veillonellaceae* were more abundant in saliva in patients with asthma than in healthy individuals [106]. Ortiz et al. observed that oral dysbiosis, a decrease in commensal bacteria, such as *Neisseria*, and an increase in opportunistic anaerobes, including *Veillonella*, occurred with the progression of periodontal disease [107]. The next-generation sequencing study showed alterations in the microbial composition of dental biofilms in the allergic (asthma and other allergic diseases) group compared to the control group. Furthermore, periopathogenetic bacteria *F. nucleatum* was enriched in the allergic group [108].

Some studies found that children with asthma had reduced salivary flow and poorer gingival condition than the healthy control group [24,109,110]. Significant differences were also observed in the saliva flow rate between adolescent patients with and without asthma [111]. Similar findings were obtained in a study examining young adult patients [112].

A low stimulated salivary flow rate coincided with a higher degree of periodontal inflammation (expressed as a higher periodontal status index) in adult patients with asthma [113,114]. Reduced salivary flow rates (when stimulated and unstimulated) and salivary buffer capacities (when unstimulated) in patients with asthma were also confirmed in a meta-analysis [115].

#### 3.2.2. Beta-2 Agonists

Among the inhaled medications, beta-2 agonists were reported to have had numerous effects on oral health. For instance, Ryberg et al. reported that patients with asthma treated with beta-2 adrenoceptor agonists showed a lower secretion rate of saliva (both whole saliva and parotid saliva) compared to healthy individuals [116,117]. They also confirmed similar findings when using terbutaline or salbutamol, commonly utilized short-acting beta-2 adrenoceptor agonists for asthma exacerbation [118].

Animal studies demonstrated that chemical sympathectomy suppressed periodontal disease and promoted bone regeneration, suggesting that sympathetic nerve stimulation may play a role in periodontal disease progression [119,120]. Gruber et al. found that beta 2-receptor agonists, including salbutamol, a selective beta 2 adrenergic-receptor agonist, inhibited the proliferation of gingival and periodontal ligament fibroblasts [121]. Beta-receptor stimulation with isoproterenol promoted alveolar bone loss in induced periodontitis in rats [122]. In a study by Okada et al., the administration of beta-blocker propranolol inhibited osteoclast differentiation and ameliorated the alveolar bone loss caused by *P. gingivalis* infection in Sprague–Dawley rats [123]. Rodrigues et al. also showed that low doses of propranolol attenuated bone resorption by suppressing osteoclast differentiation and inflammatory mediator production [124]. Collectively, these results suggest that beta-adrenergic signalling may accelerate periodontal disease.

#### 3.2.3. Inhaled Corticosteroids

Although inhaled steroids are one of the most important drugs in the treatment of asthma, various adverse effects on the oral cavity have also been reported due to their mechanisms of action. In the 1990s, Hyyppä et al. found that children with asthma who received ICS had more severe gingivitis than those with asthma who received disodium cromoglycate [125]. A cross-sectional study in India found that children with asthma aged 8–15 years showed higher PI and community periodontal index scores than healthy individuals [126]. Moreover, the dose and duration of taking ICS were associated with increased severity of periodontal disease. Among patients with asthma, ICS users had a significantly higher risk of periodontal disease than non-corticosteroid users, with an adjusted HR of 1.12 in a claims database study of national health insurance in Taiwan [26]. Combination treatment with long-acting beta-2 agonists and ICS for a month reduced the salivary flow rate in a single-blind study [127]. Bone mineral density in the mandible can be reduced by ICS, which can lead to poor periodontal health by weakening the supporting tissues of the teeth [128,129]. Defensin in the saliva possesses antimicrobial activity as well as immunomodulatory properties, such as wound healing, tissue regeneration, and elimination of inflammation [103]. Moosavi et al. measured salivary defensin levels before and after fluticasone inhalations in 17 children with asthma. They found that beta-defensin 2 concentrations in the saliva decreased after inhalation treatment and indicated that this may have led to poor oral hygiene [130].

Salivary IgA may also contribute to the maintenance of oral hygiene [99]. Patients with aggressive periodontitis [131] or generalised early onset periodontitis [132] showed statistically significantly lower concentrations and secretion rates of total salivary IgA, compared with the age- and sex-matched groups with healthy periodontium. Chang et al. showed that IgA-deficient mice revealed increased percentages of *Aggregatibacer*, *Actinobacillus*, and *Prevotella* in their saliva, and a higher level of alveolar bone loss, indicating that IgA acted protectively against periodontal disease [133]. Fukushima et al. speculate that ICS may decrease total salivary IgA levels and that patients with lower total salivary IgA levels may be more susceptible to oral infections, including candidiasis [134].

#### 3.2.4. Anticholinergics and Theophylline

Anticholinergics, such as tiotropium and ipratropium, are used for asthma treatment as inhaler medicine. A study including 1945 participants from the Northern Finland Birth Cohort indicated that anticholinergic burden was significantly associated with the number of teeth with dental plaque. The anticholinergic effect on oral hygiene is postulated to result from decreased salivary secretion. Hyposalivation is thought to promote plaque formation by decreasing oral bacterial clearance, increasing bacterial adhesion to hard and soft tissues, and decreasing the antimicrobial activity of saliva [135]. An experimental model with white Wistar rats showed that the oral administration of anticholinergics stimulated the periodontal inflammatory process and augmented the proinflammatory effects of hyaluronidase [136]. Although the degree of the effect of inhaled medications is unknown, inhaled anticholinergics can also theoretically deteriorate oral hygiene. On the contrary, only a few patients appeared to complain of dry mouth in the clinical trials using tiotropium [137,138]. Theophylline was a commonly used drug for asthma treatment in the past. The administration of theophylline promoted osteoclastic bone resorption in the teeth of rats [139]; however, this agent is not currently recommended for asthma treatment in the report of the Global Strategy for Asthma [6].

#### 3.2.5. Leukotriene-Receptor Antagonist

Leukotriene-receptor antagonists (LTRAs) play an adjunctive role in asthma therapy as an add-on to ICS and beta-2 agonists. Several reports have examined the association between LTRAs and oral diseases. In a rat model of experimental ligature-induced periodontitis, montelukast significantly reduced alveolar bone loss and gingival myeloperoxidase and increased Runt-related transcription factor 2 expression, indicating that the drug might alleviate periodontal inflammation [140]. Montelukast also exhibited inhibitory effects on osteoclast formation in a murine bone loss model [141]. Zafirlukast and its derivatives displayed antibacterial activity against the periodontal pathogen *P. gingivalis* [142,143,144]. However, there are limited reports showing an association between LTRAs and periodontal diseases in humans; therefore, the relationship needs to be further investigated.

#### 3.2.6. Discrepancies in the Data

In a study of 140 children with asthma, neither the duration of illness and medication use nor the severity of asthma were associated with dental caries, gingival bleeding, or PI [145]. Doğan et al. found no statistically significant differences between sugar-containing and sugar-free inhalers, duration of ICS use, inhaler-spacer device use, mouthwash use after inhaler administration, and plaque/gingival index [146]. Brigic and colleague assessed the oral health condition and stimulated saliva production in children with asthma aged 7–14 years. The results showed that inhaled anti-asthma medication did not induce a decrease in saliva production [147]. A nationwide population-based study in Korea demonstrated that the diagnosis of asthma was associated with periodontitis, whereas the frequency of periodontitis was lower in patients who regularly used anti-asthma medications than in those who did not receive regular treatment. Moreover, this association was not significant when treatment with anti-asthma medications was provided on an “as needed” basis, although the database lacked information on the details of the medications [15]. Scarabelot et al. investigated the effect of high and low doses of ICS in a rat model for periodontal disease. They found that only the repeated administration of low-dose ICS could reverse the effects of periodontal disease with respect to nucleotide hydrolysis, and speculated on the protective role of low-dose ICS against chronic inflammation induced by periodontal disease [148]. The influence of ICS on periodontal tissues may depend on the dose and duration of treatments.

Sharma et al. investigated the anti-inflammatory effects of salmeterol, a beta-2 agonist used as a treatment for asthma and COPD in mouse macrophages and a human monocyte cell line. They showed that salmeterol suppressed the production of inflammatory cytokines induced by lipopolysaccharide (LPS) from *P. gingivalis*, indicating that this drug may be useful in the treatment of periodontal disease [149].

### 3.3. The Impact of Asthma on Oral Diseases

There exists some evidence that asthma itself is considered a risk factor for increased oral diseases. In patients with asthma, enamel formation may be impaired due to the reduced oxygen supply that activates the ameloblasts, resulting in enamel loss and the reduced resistance of the teeth to acid [150]. Asthma in the early years of life has an impact on tooth formation and can potentially participate in the development of hypomineralised enamel lesions [151]. Regarding periodontal disease, hypoxia and subsequent reoxygenation have been reported to enhance alveolar bone resorption in human periodontal ligament cells [152]. In a rat model with periodontitis, it was shown that a chronic intermittent hypoxic condition could impair periodontal bone formation by decreasing the osteogenic markers RUNX2 and MDM21 [153].

Some patients with asthma exhibit increased concentrations of fractional exhaled nitric oxide (F_E_NO) due to airway inflammation [154]. Although low concentrations of nitric oxide are involved in important physiological functions, excessive and unregulated nitric oxide synthesis is detrimental to cells [155]. In water, nitric oxide is converted to nitric acid, and there is an increased risk of the demineralization of the hard tissues of the teeth because the oral cavity is typically quite moist. Sköld et al. speculated that this mechanism may be involved in dental caries and dental erosion [150]. Additionally, an increased level of exhaled nitric oxide may be associated with various oral disease, including periodontal diseases [156]. Numerous studies suggest a link between nitric oxide and a poor periodontal status in both human and animal models [157,158,159,160,161,162,163,164]. A meta-analysis conducted by Chen et al. found a significant increase in nitric oxide levels in the saliva of patients with chronic periodontitis compared with periodontally healthy individuals [165]. Although nitric oxide is also produced in inflamed oral tissues, it cannot be ruled out that the increased nitric oxide associated with asthma may contribute to the progression of periodontal disease.

On the contrary, Zhao et al. did not find a positive association between F_E_NO levels and the sulcus bleeding index categories or oral inflammation levels in non-asthmatic children and adolescents [166]. In addition, Silva et al. and Fukada et al. showed that the selective inhibition of inducible nitric oxide synthase promoted bone resorption, suggesting the protective role of nitric oxide against alveolar bone loss [167,168]. Aurer et al. observed an inverse association between nitric oxide levels and the severity of periodontal disease [169]. The effect of increased F_E_NO due to asthma on periodontal disease requires further study.

The behaviour of patients may also affect their oral condition. Mouth breathing habit is sometimes observed in individuals with asthma. Kairaitis et al. investigated the breathing patterns of symptomatic and asymptomatic patients with asthma and healthy individuals. According to this study, during acute exacerbations of asthma, the patient switched to oronasal breathing from simple nasal breathing. Patients with asthma, in contrast to the healthy individuals, switched to oronasal breathing from nasal breathing when wearing a face mask, regardless of the degree of bronchoconstriction [170]. A community-based cohort study including 9804 general citizens in Japan unveiled that mouth breathing was reported by 30% of the adults with asthma, which was higher than that of 17% in the general population. The study also found that mouth breathing in non-asthmatic individuals was a risk for sensitization to house dust mites, increased blood eosinophil counts, and decreased lung functions after an adjustment for AR [171]. The frequency of mouth breathing habits appeared to be even higher in young adults [112]. Another study found that mouth breathing, in addition to nasal obstruction, was more common in children with asthma than in healthy volunteers [172]. A positive correlation was found between mouth breathing (especially during sleep) and allergic diseases, including asthma, in schoolchildren [173]. People with asthma use the oral route as an alternative breathing method because they need to inhale larger volumes of air due to the airflow obstruction caused by asthma, and occasionally due to the nasal obstruction caused by concurrent AR [174]. A meta-analysis suggested an association between mouth breathing and asthma in children and adolescents (OR, 2.46) and in adults (OR, 4.60) [175]. Mouth breathing habit has a negative impact on the periodontal health in adult patients with asthma [19]. As mouth breathing continues, saliva evaporates due to the changes in humidity in the oral cavity. Consequently, saliva is unable to maintain its homeostatic functions of filtering foreign substances and controlling the humidity, temperature, and pH, which is thought to be one of the reasons for the deterioration of oral hygiene [176,177]. Mouth breathing at rest was linked to an increased level of dental plaque and the inflammation of the gingiva in schoolchildren [178]. It has also been suggested that mouth breathing may attenuate the effectiveness of scaling and root planning treatment in patients with chronic periodontitis [179].

### 3.4. Negative Effects of Oral Diseases on Asthma

**Figure 1:**
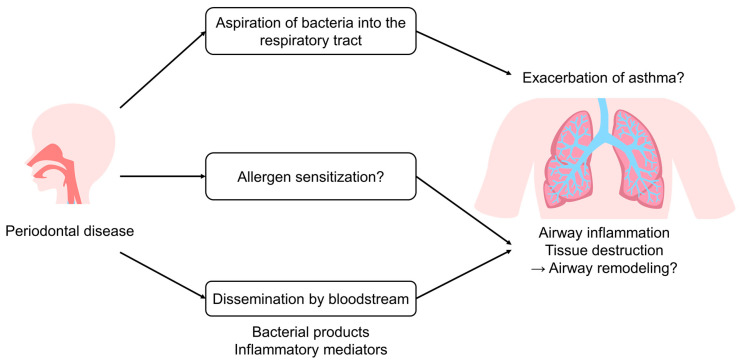
Potential pathways through which periodontal disease may affect asthma.

Several hypotheses have been proposed regarding the pathways by which oral diseases may affect asthma. Some of the representative ones are highlighted here (Figure 1).

Microaspiration is common in healthy adults. This is supported by the fact that there is an overlap between common oropharyngeal and lung bacteria, whereas the nasal and lung microbiota share fewer similarities [2]. The aspiration of bacteria into the lower respiratory tract can affect the clinical course of asthma. The microbiota involved in the exacerbation of asthma include *Streptococcus pneumoniae*, *Haemophilus influenzae*, and *Moraxella* species in children [180]. In adults, the bacterial colonisation of the lower respiratory tract was also associated with a history of exacerbations in the past year [181].

In contrast, mechanisms have been postulated by which substances of oral origin can affect distant organs via the bloodstream. Among them, matrix metalloproteinases (MMPs) are one of the most investigated factors. Through hematogenous spread, MMPs can contribute to the destruction of the structural protein of the respiratory tract, leading to airway remodelling [182]. The expression of MMP-9 has been reported to be up-regulated in patients with asthma and COPD [183]. A study by Carneiro et al. revealed the increased expression of MMP-9 in epithelialized and non-epithelialized apical periodontitis lesions when compared to healthy periapical ligaments. The number of MMP-9 positive cells and their ratio to total cells were higher in non-epithelialized than epithelialized lesions [184]. These results indicate that MMP-9 may be involved in the degradation of the extracellular matrix in apical periodontitis lesions. Another study found a positive correlation between periodontal pocket depth and MMP-9 concentration in patients with moderate or severe periodontitis, and also between the concentration of MMP-9 in the oral fluid and the number of affected teeth [185]. Kim et al. reported that the levels of salivary MMP-9 and S100A8 were associated with periodontitis and their usefulness in diagnosing the disease [186]. A meta-analysis also showed statistically significant differences in MMP-9 levels in serum and gingival crevicular fluid between patients with periodontitis and periodontally healthy individuals [187]. Suzuki et al. showed that periodontopathic bacteria *F. nucleatum* induced the expression of MMP-9 in mouse lung and bronchoalveolar lavage fluid. The production of the MMP-9 protein and mRNA was also induced by *F. nucleatum* in a density-dependent manner in A549 cells. The authors suggest that *F. nucleatum* aspirated into the lungs may cause the onset and progression of pulmonary disease through the expression of MMP-9 [188]. Zhang et al. demonstrated that in the human immortalized oral epithelial cell/human gingival fibroblast co-culture model, the stimulation of *P. gingivalis* lipopolysaccharide promoted MMP-9 activation. Moreover, the inhibition of MMP-9 suppressed extracellular matrix degradation in the same model [189]. Collectively, these findings suggest that oral disease may lead to the progression of respiratory disease, including asthma, via the hematogenous spread of MMP-9 from the periodontal tissues.

When IgE binds to the high-affinity IgE receptor on mast cells and basophils, the cells are activated and release inflammatory mediators through degranulation. This leads to chronic inflammation and the narrowing of the airways, increased airway hyperresponsiveness, and bronchospasm in asthma [190]. Hyyppä conducted a study that examined the relationship between asthma and periodontal disease based on salivary concentrations of IgE and histamine. The results showed that IgE, histamine, and lysozyme concentrations in patients with asthma and periodontal disease were considerably higher than those in individuals without periodontal disease or asthma. The author speculated that IgE-mediated hypersensitivity reactions may occur in the gingival tissue [191]. Hara et al. also confirmed the presence of IgE-bearing cells in the gingival tissue collected from patients with chronic periodontitis and a significantly higher ratio of IgE-bearing cells to total inflammatory cells compared to the healthy subjects [192]. Higher serum IgE levels were also reported in patients exhibiting asymptomatic dental necrosis compared to the healthy volunteers. In this study, it was hypothesized that when allergens in necrotic pulp tissue were recognized by antigen-presenting cells, it was presented to T lymphocytes, which in turn differentiated B lymphocytes into plasma cells and produced antigen-specific IgE, thereby leading to IgE-mediated sensitization in patients with periodontal disease [193]. Han and colleagues exhibited an inverse association between serum total IgE and the number of natural teeth using the Korean national database. They speculated that the sensitization to oral pathogens may be related to destructive periodontal disease [194]. Although these studies are originally indicative of the allergic nature of periodontal diseases, it is also of interest to understand how allergen sensitization in periodontal tissues may affect the onset or progression of asthma.

In an experiment by Card et al. where mice were treated with *P. gingivalis* infection and ovalbumin sensitization with different orders of administration, serum IgE levels were elevated in the model where the infection initiated either before or after ovalbumin sensitization, compared to the sensitization-only group. The authors suggested an important immunomodulatory effect of this bacterium [195]. A similar finding was confirmed by another study that the administration of LPS from *P. gingivalis* to neonatal mice increased serum IgE levels at maturity, while LPS from *A. actinomycetemcomitans* or *Escherichia coli* did not [196]. However, in the study by Card et al., airway hyperresponsiveness did not alter or was even reduced depending on the timing of infection, despite elevated serum IgE levels [195], and how IgE-mediated reactions due to periodontal disease relate to asthma is a subject for future investigations.

## 4. Conclusions and Future Perspective

**Figure 2:**
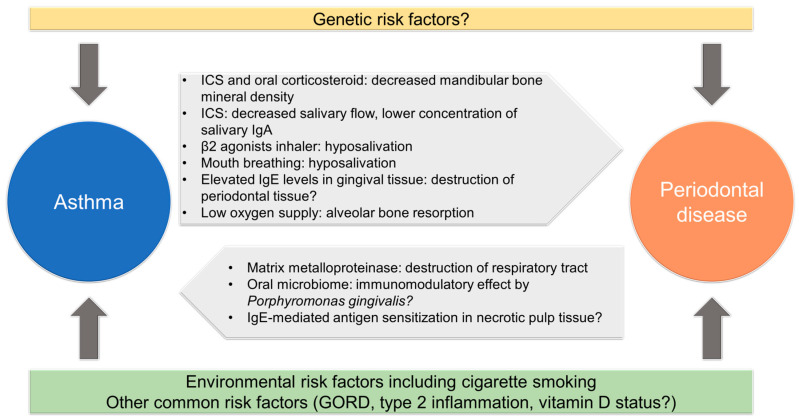
Possible factors linking periodontal disease and asthma. GORD, gastro-oesophageal reflux disease; ICS, inhaled corticosteroid; Ig, immunoglobulin.

Numerous studies have indicated a link between oral disease and asthma, as summarized in Figure 2, although some have yielded conflicting results. Various factors are involved in this controversy, including the background of the patients studied, differences in medications, how the outcomes are measured, the availability of appropriate inhalation techniques (including gargling and mouth washing), and the proper statistical treatment of confounding factors, such as smoking, economic status, and tooth brushing behaviour. Most studies to date are retrospective, and interventional studies, e.g., those investigating whether the treatment of oral disease reduces disease development or improves disease control in asthma, are limited. The biological mechanisms that underlie the relationship have also not been fully elucidated.

Impact of periodontal disease on the QOL in patients with asthma is also an area for further study. Although there are reports investigating the oral-related quality of life in patients with asthma complicated by periodontal disease [17], there are few reports examining the overall QOL using the respiratory disease-related QOL assessment (e.g., St George’s Respiratory Questionnaire) or asthma-related QOL assessment (Asthma Quality of Life Questionnaire, Asthma Bother Profile, Asthma Impact Survey, etc.) [197]. The association between periodontal disease and asthma should be examined from various viewpoints, including the development of disease, frequency of exacerbations, decline in pulmonary function, and deterioration of QOL.

Changes in therapeutic agents should also be considered. It is hard to compare studies from the time when beta-2 agonists were the mainstay of treatment with those from the time when the guidelines recommended ICS as the principal therapeutic agent. The impact of recently introduced ICS and beta-2 agonists, in addition to biologics medication, needs to be examined. Most clinical studies on the association between inhaled medications and oral hygiene focus on children to middle-aged individuals [102]; therefore, it is necessary to examine this topic in the older adult population as well. The relationship between the two diseases is more complex in the older population, as they are more likely than younger people to be affected by ageing and smoking in the development of periodontal diseases, both of which can be confounding factors. Moreover, a recent study reported that maternal periodontal disease had protective effects on the development of asthma in the offspring in rats [198], and such a transgenerational impact should also be evaluated. Future research to resolve these issues is warranted.

## Figures and Tables

**Table 1:** Observational studies that support the detrimental association between periodontal disease and asthma.

Author (Year)	Reference	Participants	Age (Years)	Study Design Country	Periodontal Parameters	Covariate Adjustment	Summary of Main Results
Abe et al., 2020	[11]	9098 University students (1782 females and 7316 males)	18.3	Cross-sectional Japan	Self-reported bleeding while brushing their teeth	Sex and comorbidities (pollinosis, food/drug allergy, inhaled antigen allergy, atopic dermatitis, allergic rhinitis, otitis media/externa, sinusitis, respiratory infectious diseases, pneumothorax/mediastinal emphysema, and asthma/cough-variant asthma)	Self-reported gum bleeding was associated with asthma/cough-variant asthma (OR = 1.303, 95% CI = 1.091–1.556, *p* = 0.003)
Wee et al., 2020	[14]	136,027 Participants selected from the Korea Youth Risk Behaviour Web-based Survey 2014 to 2015 (66,484 females and 69,543 males)	15.0 (standard error = 0.02)	Cross-sectional South Korea	Self-reported oral symptoms during the past 12 months	Age, sex, economic level, region of residence, parents’ educational level, obesity, smoking, alcohol intake, physical activity, history of other allergic disease, and dental health-related behaviours	Poor oral health was significantly correlated with the prevalence of asthma (adjusted OR = 1.48, 95% CI = 1.34–1.63, *p* < 0.001)
Lee et al., 2017	[15]	5976 Participants selected from the Sixth Korean National Health and Nutrition Examination Survey (KNHANES) in 2014 (3422 females and 2554 males)	51.4	Cross-sectional South Korea	CPI	Age, sex, marital status, health insurance type, presence of private health insurance, level of education, and lifestyle factors reflecting the Korean health insurance structure	Current asthma condition was associated with periodontitis (adjusted OR = 5.36, 95% CI = 1.27–22.68, *p* < 0.05)
Wee et al., 2020	[16]	227,977 Participants from the Korean Community Health Survey 2015 (125,380 females and 102,597 males)	Range: 19 and older	Cross-sectional South Korea	Self-reported oral health status, periodontal status, frequency of tooth brushing, and scaling history within the past 12 months	Age, sex, economic level, education level, region of residence, smoking, alcohol, obesity, subjective health status, stress level, and physical activity	Poor oral health status was associated with asthma, with an adjusted OR of 1.19 (95% CI = 1.07–1.33, *p* = 0.002)
Brasil-Oliveira et al., 2020	[17]	125 Patients with severe asthma, *n* = 40 (34 females and 6 males); patients with mild-to-moderate asthma, *n* = 35 (30 females and 5 males); participants without asthma, *n* = 50 (24 females and 26 males)	Severe asthma group, 51.8 ± 10.8; mild-to-moderate asthma group, 42.5 ± 14.2; no-asthma group, 48.2 ± 12.4	Cross-sectional Brazil	OHIP-14, SF-36 version 2, and WAI	–	Patients with severe asthma had lower scores on the OHIP-14 domain than patients without asthma (*p* < 0.001) and those with mild-to-moderate asthma (*p* = 0.013). SF-36 version 2 physical component summary scores were lower in the individuals in the severe asthma group compared with no asthma groups (*p* < 0.001) The WAI was also lower among the individuals in the severe asthma group than among those in the mild-to-moderate asthma (*p* = 0.022) and no asthma groups (*p* < 0.001)
Shen et al., 2017	[26]	96,030 Participants selected from the National Health Insurance of Taiwan (48,105 females and 47,925 males) 19,206 patients with newly diagnosed asthma from 2000 through 2010, were included	The asthma cohort, 41.5 ± 25.9; the comparison cohort, 41.3 ± 25.7	Retrospective cohort Taiwan	Occurrence of periodontal diseases (ICD-9-CM code 523.0 to 523.9) Patients with a history of gingival and periodontal diseases at baseline were excluded	Age, sex, monthly income, urbanization level, allergic rhinitis, atopic dermatitis, chronic sinusitis, GORD, obesity, smoking-related diseases, alcohol-related diseases, diabetes, osteoporosis, depression, anxiety, tooth loss, and caries	The overall incidence of periodontal disease was higher in the asthma group (38.6 per 1000 person-years vs. 32.5 per 1000 person-years), with an adjusted HR of 1.18 (95% CI = 1.14–1.22) Frequent emergency room visits and hospitalisations were associated with a higher risk of developing periodontal diseases The use of ICS resulted in a significantly increased risk of periodontal diseases (adjusted HR = 1.12, 95% CI = 1.03–1.23), while systemic corticosteroid did not (adjusted HR = 1.04, 95% CI = 0.96–1.14)
McDerra et al., 1998	[18]	249 Case (children with asthma), *n* = 100; control (children without asthma), *n* = 149 The control group was matched for age, sex, race, and socioeconomic status	Range: 4–16	Case-control England	Gingivitis and dental plaque scores	–	Children with asthma had higher gingivitis scores than the control participants (*p* < 0.01) Children with asthma aged 4–10 years had higher plaque scores than the control participants (*p* < 0.05)
Gomes-Filho et al., 2014	[19]	220 Case (patients with asthma), *n* = 113 (92 females and 21 males); control (participants without asthma), *n* = 107 (92 females and 15 males)	Case, 46.8 ± 11.2; control, 43.6 ± 14.4	Case-control Brazil	PD, CAL, BOP, and PI	Age, education level, osteoporosis, smoking habit, and BMI	Periodontitis was associated with severe asthma (adjusted OR = 4.82, 95% CI = 2.66–8,76)
Soledade-Marques et al., 2018	[20]	260 Case (patients with severe asthma), *n* = 130 (104 females and 26 males); control (participants without asthma), *n* = 130 (113 females and 17 males)	48.2 ± 14	Case-control Brazil	PD, CAL, BOP, and PI	Age, schooling level, family income, household density, osteoporosis, hypertension, diabetes, smoking habit, and BMI	The association between periodontitis and severe asthma was indicated by logistic regression models adjusted for nine different potential confounding variables (adjusted OR = 3.01–3.25, 95% CI = 1.70–5.76, all *p* < 0.01)
Lopes et al., 2020	[21]	457 Case (patients with asthma), *n* = 220 (180 females and 42 males); control (participants without asthma), *n* = 237 (206 females and 31 males)	Case, 51 ± 12; control, 45 ± 11	Case-control Brazil	PD, CAL, BOP, and PI	Age, family income, hypertension, current smoking habits, BMI, and mouth breathing behaviour	There was a statistically significant positive correlation between periodontitis and severe asthma (adjusted OR = 4.00, 95% CI = 2.26–7.10)
Khassawneh et al., 2019	[22]	260 Case (patients with asthma), *n* = 130 (74 females and 56 males); control (participants without asthma), *n* = 130 (77 females and 53 males)	Case, 46.43 ± 12.24; control, 44.18 ± 11.85	Case-control Jordan	PI, GI, PD, CAL, gingival recession, and BOP	Age, sex, income, highest education, residency, and smoking	Patients with asthma were more likely to have periodontitis than the controls (adjusted OR = 2.91, 95% CI = 1.39–6.11, *p* = 0.005). Patients on oral corticosteroids had a higher risk of periodontitis and CAL ≥ 3 mm compared with those on ICS
Bhardwaj et al., 2017	[23]	100 (46 females and 54 males) Case (patients with asthma), *n* = 50; control (participants without asthma), *n* = 50	Females, 41.62; males, 38.7	Case-control India	PI, GI, PBI, calculus index, and CAL	–	Patients with asthma had worse scores on these parameters than individuals without asthma, suggesting a poorer periodontal condition
Mehta et al., 2009	[24]	160 Case (patients with asthma), *n* = 80; control (participants without asthma), *n* = 80 The control group was matched for age, sex, and socioeconomic status	Case, 17.4 ± 4.3; control, 17.2 ± 4.2	Case-control India	Modified Quigley–Hein plaque index and modified GI	–	The mean plaque index and gingival index scores were higher in patients with asthma (both *p* < 0.001), indicating a poorer periodontal status
Moeintaghavi et al., 2022	[25]	140 Case (newly diagnosed patients with asthma), *n* = 70 (38 females and 32 males); control (healthy participants), *n* = 70 (36 females and 34 males)	Case, 37.7 ± 9.0; control, 38.3 ± 9.7	Case-control Iran	PD, CAL, GI, and PI	–	Patients with asthma had significantly higher PI, GI, PD, and AL scores than healthy individuals (*p* < 0.001).
Gómez Real et al., 2016	[27]	13,409 Participants selected from the Respiratory Health in Northern Europe III cohort (female, 53%)	52	Population-based cohort Northern European centres (Norway, Sweden, Denmark, Iceland, and Estonia)	Self-reported bleeding while brushing their teeth, CPI in sub-population (*n* = 261)	Age, sex, smoking, educational level, study centre, cardio-metabolic disease, frequency of tooth brushing, GORD, nasal congestion, early life developmental factors (mother’s age when giving birth to the participants, parental smoking, severe respiratory infections in childhood, and fruit intake in childhood), and asthma medication	Gingival bleeding was significantly associated with three or more asthma symptoms (OR = 2.58, 95% CI = 2.10–3.18), asthma (1.62 [1.23–2.14]), and self-reported COPD (2.02 [1.28–3.18]). A dose–response relationship was found between respiratory outcomes and frequency of gingival bleeding (three or more symptoms: gingival bleeding sometimes 1.42 [1.25–1.60] and often/always 2.58 [2.10–3.18])

AL, attachment loss; BMI, body mass index; BOP, bleeding on probing; CAL, clinical attachment loss; CI, confidence interval; CPI, community periodontal index; COPD, chronic obstructive pulmonary disease; GI, gingival index; GORD, gastro-oesophageal reflux disease; ICS, inhaled corticosteroid; OHIP, oral health impact profile; OR, odds ratio; PBI, papillary bleeding index; PD, probing depth; PI, plaque index; SF, Short-Form 36-Item Health Survey; WAI, work ability index. Ages are expressed as mean ± standard deviation unless otherwise specified.

**Table 2:** Investigations exploring the impacts of periodontal treatment on asthma-related outcomes.

Author (Year)	Reference	Study Design Country	Participants	Age (Years)	Periodontal Treatment	Measured Outcome	Summary of Main Results
Enomoto et al., 2023	[33]	Cross-sectional Japan	27,185 Participants selected from the panellists of a Japanese Internet research company to represent the Japanese population regarding age, sex, and residential prefecture Patients with asthma, *n* = 677 (male, 49.2%)	Range: 15–79 Patients with asthma: 50.1 ± 17.3	The impact of the discontinuation of dental treatment during the COVID-19 pandemic on disease exacerbation was investigated	Self-reported exacerbation of asthma	The absence of dental treatment was a significant factor in the exacerbation of asthma (*p* = 0.0094) after the adjustment of covariates (age, sex, smoking, living situation, homeownership status, educational background, and income)
Shen et al., 2017	[32]	Propensity-matched cohort Taiwan	Participants selected from the National Health Insurance claims data of Taiwan Periodontal treatment group: individuals with asthma comorbid with periodontal disease, *n* = 4771 Control group: individuals with asthma not complicated by periodontal disease, *n* = 4771	Periodontal treatment group: 61.9 ± 16.6 Control group: 62.2 ± 16.6	Periodontal treatment group: subgingival curettage (scaling and root planning) and periodontal flap surgery	Adverse respiratory events (acute asthma exacerbation, pneumonia, acute respiratory failure, hospitalisation, and ICU admission)	Overall rates of hospitalisation for respiratory adverse events (adjusted IRR = 0.84, 95% CI = 0.78–0.92) and ICU admissions (adjusted IRR = 0.88, 95% CI = 0.79–0.99) were lower in the periodontal treatment group compared with the control group after the adjustment of covariates (age, sex, monthly income, urbanization level, comorbidities, and level of asthma therapy)
Pambudi et al., 2008	[34]	Randomized controlled trial Indonesia	36 children with asthma Intervention group: *n* = 18 (female, *n* = 8; male, *n* = 10) Control group: *n* = 18 (female, *n* = 9; male, *n* = 9)	Range: 6–12 Intervention group: 9.2 ± 2.3 Control group: 8.9 ± 2.3	Intervention group: dental plaque removal by oral biology dentist and guide to perform an individual oral health care Control group: observation without intervention	Dental plaque culture, blood eosinophil count, pulmonary function test, and 4-point scale asthma score	Plaque analysis of participants who underwent dental treatment showed a significant reductions in the number of microbial colonies (×10^8^ cfu/mL, mean ± SD: pre = 5.0 ± 2.0; post = 3.2 ± 2.1; *p* < 0.01) and Gram-negative bacilli, while no significant changes were observed in the control group Decreases in airway reversibility, asthma symptoms, and blood eosinophil counts were also observed in the treatment group
Nelwan et al., 2019	[35]	Randomized controlled trial Indonesia	10 participants with gingivitis and a positive skin-prick test to house dust mites Intervention group: *n* = 5 (female, *n* = 3; male, *n* = 2) Control group: *n* = 5 (female, *n* = 3; male, *n* = 2)	Range: 6–16 Intervention group: 10.2 ± 3.9 Control group: 9.8 ± 2.4	Intervention group: SRP and standard allergic treatments Control group: standard allergic treatments only	Serum IgE and IgG4	The intervention group showed more marked improvements (*p* < 0.00) in IgE ([pg/mL, mean ± SD]: pre = 99.84 ± 2.16, post = 80.03 ± 1.65) and IgG4 (pre = 28.62 ± 3.88, post = 18.05 ± 2.38) levels than the control group (IgE: pre = 139.42 ± 1.49, post = 138.48 ± 1.45; IgG4: pre = 38.66 ± 1.85, post = 38.75 ± 1.87)

cfu, colony forming unit; CI, confidence interval; COVID-19, coronavirus disease 2019; ICU, intensive care unit; Ig, immunoglobulin; IRR, incidence rate ratio; SD, standard deviation; SRP, scaling and root planning. Ages are expressed as mean ± standard deviation unless otherwise specified.

**Table 3:** Studies that do not support an association between periodontal disease and asthma or suggest a protective association.

Author (Year)	Reference	Participants	Age (Years)	Study Design Country	Periodontal Parameters	Covariate Adjustment	Summary of Main Results
Shulman et al., 2003	[36]	1596 Participants selected from the Third National Health and Nutrition Examination Survey (NHANES III) 1988–1994 (849 females and 747 males)	Range: 13–17	Cross-sectional USA	BOP, subgingival calculus, supragingival calculus, PD greater than or equal to 3 mm, and loss of periodontal attachment greater than or equal to 2 mm	Income, sex, race (White/non-White), exposure to potentially xerogenic drugs (antihistamines, corticosteroids, and inhalers), smoking, and dental examination within the past year	Neither asthma nor the cumulative use of anti-asthmatic medication was significantly associated with periodontal indices
Shah et al., 2022	[37]	10,710 Participants selected from the National Health and Nutritional Examination Survey (NHANES) 2009–2014 (5438 females and 5272 males)	Age group: 30–44, *n* = 3865 (36.1%); 45–64, *n* = 4540 (42.4%); ≥65, *n* = 2305 (21.5%)	Cross-sectional USA	CAL and PD	Age, race/ethnicity, sex, education, income, BMI, diabetes, and smoking	Patients with current asthma had lower odds of severe periodontitis, compared with individuals without asthma (adjusted OR = 0.51, 95% CI = 0.30–0.87). No statistically significant association was found between asthma and other forms of periodontitis
Chatzopoulos et al., 2021	[38]	4890 Randomly selected patients who had attended the University of Minnesota dental clinic (male, 52.7%)	54.1 ± 17.9	Retrospective chart review USA	ABL	Age, sex, smoking, and diabetes	The presence of asthma seemed to be protective against ABL (adjusted OR = 0.695, 95% CI = 0.564–0.857)
Lemmetyinen et al., 2021	[39]	1394 patients with asthma (identified from the Drug Reimbursement Register of the Finnish Social Insurance Institution) 2398 individuals without asthma (identified from the Population Register)	Asthma group: ≤59, *n* = 1506 (62.8%); 60–69, *n* = 699 (29.1%); 70–79, *n* = 166 (6.9%); ≥80, *n* = 27 (1.1%) No-asthma group: ≤59, *n* = 893 (64.1%); 60–69, *n* = 401 (28.8%); 70–79, *n* = 84 (6.0%); ≥80, *n* = 16 (1.1%)	Population-based matched cohort (sex, age, and area of residence matched) Finland	Dental diseases (ICD-10 code: K00–K14, including tooth decay, chronic apical periodontitis, sialadenitis, and diseases of periodontal tissue)	Smoking, education level, and BMI	Dental diseases were not significantly associated with adult asthma (adjusted HR = 1.40, 95% CI = 0.93–2.12)
Hozawa et al., 2022	[40]	42,685 Participants selected from the Japanese insurance claims database (May 2014–April 2019) Patients with asthma who have experienced exacerbation, *n* = 5844; patients with asthma who have never experienced an exacerbation, *n* = 36,841	Exacerbation group, 44.3 ± 12.4; no-exacerbation group, 43.6 ± 12.8	Retrospective cohort Japan	Periodontal diseases (ICD-10 code: K053)	Age, sex, frequency of pulmonary tests, use of ICS/SABA/OCS, and other complications (allergic rhinitis, chronic sinusitis, atopic dermatitis, acute airway disease, COPD, chronic bronchitis, GORD, hypertension, diabetes, and dyslipidaemia)	Periodontal disease was associated with a decreased risk of asthma exacerbations (adjusted HR = 0.93, 95% CI = 0.88–0.98, *p* = 0.006)
Ho et al., 2019	[41]	51,439 Participants selected from the National Health Insurance Research Database in Taiwan (28,541 females and 22,898 males)	Age group of 21–25	Population-based research Taiwan	Periodontal diseases (ICD-9-CM code: 523.3 and 523.4)	Sex, socioeconomic status, urbanization, dentofacial anomalies, disease of salivary flow, diabetes mellitus, and oesophageal reflux	After adjusting for allergic rhinitis, the association between asthma and periodontal disease was not statistically significant (RR = 1.02, 95% CI = 0.98–1.06, *p* = 0.290)
Arbes Jr et al., 2006	[44]	9385 Participants selected from the third National Health and Nutrition Examination Survey 1988– 1994 (5269 females and 4103 males)	Age group: 12–29, *n* = 3172 (33.8%); 30–49, *n* = 2831 (30.2%); 50–90, *n* = 3369 (35.9%)	Cross-sectional USA	Serum IgG antibody concentrations to periodontopathic bacteria *Porphyromonas gingivalis* and *Actinobacillus actinomycetemcomitans*	Age, sex, race–ethnicity, education level of family, census region, urbanization, serum cotinine, and BMI	Higher serum concentrations of IgG antibodies against *P*. *gingivalis* and *A*. *actinomycetemcomitans* were significantly associated with lower prevalence of asthma and/or wheezing: adjusted ORs were 0.41 (95% CI, 0.20–0.87) for asthma and 0.43 (0.23–0.78) for wheezing in *P*. *gingivalis*; 0.39 (0.17–0.86) for wheezing in *A*. *actinomycetemcomitans*
Du et al., 2006	[45]	Discovery dataset, *n* = 3700; validation dataset, *n* = 4453 Participants selected from the third National Health and Nutrition Examination Survey 1988–1994 (4357 females and 3796 males)	Discovery dataset, 60.86 ± 13.70; validation dataset, 60.69 ± 13.83	Cross-sectional USA	Serum IgG antibodies against 19 periodontal pathogenic species	Age, sex, race/ethnicity, poverty income ratio, educational attainment, healthy eating index, BMI, smoking, dental visits, and systemic diseases (arthritis, heart disease, stroke, hypertension, diabetes, and cancer)	Lower levels of antibodies to periodontal pathogens were associated with a higher risk of asthma (adjusted OR = 1.820, 95% CI = 1.153–2.873) and wheezing (adjusted OR = 1.550, 95% CI = 1.095–2.194) in the discovery dataset Consistent results were obtained in the validation dataset
Friedrich et al., 2006	[47]	2837 Participants selected from inhabitants living in Pomerania area (female, 50.6%)	Range: 20–59 Participants without respiratory allergies (mean ± standard error), 39.6 ± 0.2 Participants with hay fever, 35.2 ± 0.6 Participants with a house-dust-mite allergy, 34.5 ± 1.0 Participants with asthma, 37.9 ± 1.0	Cross-sectional Germany	AL	Age, sex, school education, smoking status, alcohol consumption, family history for allergies or asthma, and number of teeth	A slight inverse association was observed between asthma and AL (severity of AL: mild; OR = 1.10 [95% CI = 0.6–2.0]; moderate; OR = 0.96 [95% CI = 0.5–1.8]; severe; OR = 0.48, [95% CI = 0.2–1.0]; *p* (trend) = 0.11)
Friedrich et al., 2008	[48]	170 patients with type-1 diabetes mellitus (female, 45.9%)	Range: 40–65 Participants without respiratory allergies (mean ± standard error), 37.1 ± 1.1 Participants with respiratory allergies, 37.5 ± 2.5	Cross-sectional Germany	AL	Age, sex, smoking, and duration of diabetes	Compared with individuals with healthy periodontal condition, patients with severe periodontal condition had the lowest risk of respiratory allergies, including asthma (adjusted OR = 0.05, 95% CI = 0.01–0.35), followed by moderate AL (adjusted OR = 0.12, 95% CI = 0.02–0.60) and mild AL (adjusted OR = 0.30, 95% CI = 0.08–1.07)
Rivera et al., 2016	[46]	1315 Participants selected from San Juan Overweight Adults Longitudinal Study (male, 27.8%)	Range: 40–65	Cross-sectional Puerto Rico	BOP and PI	Age, sex, smoking status, BMI, family history of asthma, and income level	Patients with severe periodontitis were less likely to have asthma compared with participants with none/mild periodontitis (adjusted OR = 0.44, 95% CI, 0.27–0.70)
Sperr et al., 2018	[49]	3597 Case (patients seen at the Division of Conservative Dentistry and Periodontology of the University Clinic of Dentistry, Medical University of Vienna), *n* = 1199 (female, 53.5%); control (general Austrian population as assessed in the Austrian Health Survey 2006/2007, age- and sex-matched and those who were living in the same area), *n* = 2398 (female, 53.5%)	Case, 49.3 ± 12.3; control, 49.7 ± 13.0	Case-control Austria	AL, PD, papillary bleeding index, and PI	Age, sex, education, smoking, alcohol consumption, and BMI	Prevalence of asthma was significantly lower in patients with periodontitis compared with the Austrian population (1.5% vs. 5.6%, adjusted OR = 0.169, 95% CI = 0.106–0.270, *p* < 0.001)
Jiao et al., 2023	[51]	Sample size: asthma data, *n* = 462,933 (53,598 cases and 409,335 controls); periodontitis data, *n* = 198,441 (3046 cases and 195,395 controls) Data from the European population (UK Biobank)	Range: 40–73 (UK Biobank)	Two-sample Mendelian randomization analysis	Periodontal diseases (ICD-10 code: K05.30 and K05.31)	Each SNP was searched using the Phenoscanner website to ensure that there were no confounding factors, such as smoking	Asthma may be a protective factor for periodontitis (inverse variance weighted OR = 0.34, 95% CI = 0.132–0.87, *p* = 0.025). Meanwhile, no evidence was found that periodontitis was causally related to the development of asthma

ABL, alveolar bone loss; AL, attachment loss; BMI, body mass index; BOP, bleeding on probing; CAL, clinical attachment loss; CI, confidence interval; COPD, chronic obstructive pulmonary disease; GORD, gastro-oesophageal reflux disease; ICD-9-CM, International Classification of Diseases, 9th Revision, Clinical Modification; ICD-10, the International Classification of Diseases, 10th edition; ICS, inhaled corticosteroid; OCS, oral corticosteroid; OR, odds ratio; PD, probing depth; PI, plaque index; RR, relative risk; SABAs, short-acting β-agonists; SNP, single nucleotide polymorphism. Ages are expressed as mean ± standard deviation unless otherwise specified.

**Table 4:** Anti-asthma medications and possible effects on oral health.

Type of Medication	Significance in the Treatment of Asthma	Possible Impacts on Oral Health
Beta-2 agonists	Bronchodilation	Decreases salivary flow Reduces the buffering capacity of saliva Inhibits the proliferation of gingival and periodontal ligament fibroblasts Promotes alveolar bone loss in rats
Inhaled/oral corticosteroids	Anti-inflammatory effect	Decreases salivary flow Decreases salivary IgA, defensin Reduces bone mineral density in the mandible
Muscarinic antagonist	Bronchodilation	Decreases salivary flow
Leukotriene-receptor antagonist	Anti-inflammatory effect	Lowers alveolar bone loss and gingival myeloperoxidase in rats Portrays inhibitory effects on osteoclast formation in mice Antibacterial activity against the periodontal pathogen *P*. *gingivalis*
Theophylline	Bronchodilation Anti-inflammation with low dose	Activates osteoclastic bone resorption in rat teeth

IgA, immunoglobulin A; *P. gingivalis*, *Porphyromonas gingivalis*.

## Data Availability

Not applicable.
